# Analysis of Japanese consumers' attitudes toward the digital transformation of OTC medicine purchase behavior and eHealth literacy: an online survey for digital experience design

**DOI:** 10.3389/fdgth.2023.1173229

**Published:** 2023-05-24

**Authors:** Guyue Tang, Kairi Izumi, Megumi Izumisawa, Shinichi Koyama

**Affiliations:** ^1^ Graduate School of Comprehensive Human Sciences, University of Tsukuba, Ibaraki, Japan; ^2^ Department of Pharmacy, Nihon University, Chiba, Japan; ^3^ Faculty of Art and Design, University of Tsukuba, Ibaraki, Japan

**Keywords:** consumer behavior, digital health, eHealth literacy, OTC medicine, user experience, human factors

## Abstract

**Introduction:**

Since the enactment of the revised Pharmaceutical Affairs Act in Japan in 2009, self-medication practices have increased in the country. However, studies report that consumers pay little attention to the medication facts and risks indicated on the packages of over-the-counter (OTC) medicines, which could be a potential risk. Since the COVID-19 pandemic, the digital transformation of purchasing OTC medicines has progressed. As an appropriate design for the digital transformation is likely to improve consumers' literacy and them obtaining medical information, this study systematically examines Japanese consumers' attitudes toward the digital transformation of OTC medicine purchase behavior and its correlation to eHealth literacy, exploring an appropriate digital experience design in purchasing OTC medicine.

**Methods:**

Participants from the Greater Tokyo Area of Japan participated in an online survey. Consumers' current behavior and preferences in accessing OTC medicine, receiving medication guidance, and obtaining medical information were examined. eHealth literacy was assessed using the J-eHEALS. Descriptive statistics, text mining, and thematic analysis were conducted to answer research questions.

**Results:**

Over 89% of the respondents who had experience in purchasing OTC medicines preferred local pharmacies or stores rather than online purchasing, *p* < 0.001. Obtaining medicine guidance in pharmacies or stores was the main preference over other approaches, *p* < 0.001. Furthermore, most of the participants accepted selecting medicine on shelves and digital screens in-store. However, they were accustomed to using smartphones to obtain additional information at the pharmacy or drug store, *p* < 0.001; this behavior was positively correlated with eHealth literacy, *p* < 0.001.

**Conclusions:**

Japanese consumers are seeking a combination of conventional and digital behaviors for purchasing OTC medicine rather than opting for a particular method. Most consumers prefer purchasing and receiving instructions in-store while searching for additional decision-making information online. eHealth literacy is positively associated with digital behaviors of OTC medicine information acquisition but less associated with medicine purchases and selections. The hybrid digital experience design may enhance the OTC medicine purchase experience and reduce potential risks by providing appropriate information.

## 1. Introduction

Japan promotes self-health management, and self-medication practices have increased in the country since 2009 (1, 2). Although over-the-counter (OTC) medicine plays an essential role in self-mediation in Japan, a previous study has reported potential problems and risks associated with Japanese consumers' perceptions of medical information printed on medicine packages (3). For example, they are likely to pay more attention to brand names than to ingredients and directions for usage, which might cause an inappropriate choice and use of the OTC medicines (3). Additionally, other studies have shown that consumers encounter problems in purchasing medicine and reading its information (4–6), which is related to health literacy (7, 8).

On the other hand, health information technology (HIT) plays an essential role in self-health management in Japan (9). Consumers can obtain health information for self-medication and OTC medicine through Internet-based devices. However, considering health literacy in HIT (10), Japanese consumers may find it difficult to make appropriate decisions regarding health information on the Internet (11, 12). Since the emerge of COVID-19, digital transformation of the purchase of OTC medicine has been in progress. However, since an appropriate digital transformation design is likely to improve consumers' literacy and reduce the potential barriers and risks during medicine purchases and obtaining medical information, we attempt to explore an appropriate digital experience design by examining Japanese consumers' behavior and preferences in purchasing OTC medicine.

As medicine that can be purchased without a prescription, OTC medicine plays a primary role in consumer self-medication (13–16). Owing to the rapid development of HIT from a global perspective, consumers’ health behavior with regard to purchasing OTC medicine is constantly changing. As early as the year 2000, researchers proposed that combining online and offline purchasing methods may provide consumers with great convenience (17), but required empirical evidence to substantiate the claim. Subsequently, surveys on the behavior of purchasing OTC medicines showed that, on the one hand, the vast majority of consumers bought medicines at local pharmacies due to concerns about the safety of purchasing online (18, 19). On the other hand, convenience and information acquisition advantages (20) led some consumers to prefer buying online (21).

However, the COVID-19 pandemic may be a catalyst for accelerated change, not only in consumer behavior (22, 23) but also in OTC medicine purchasing and self-medication behavior (24). During this period, OTC medicine sales and self-medication rates increased in some countries (13, 25). Owing to their easy availability, consumers purchase OTC medicines for self-medication, causing significant health safety risks (26, 27). Inappropriate self-medication has led to unchecked medicine abuse and dependence (28), culminating in a growing public health issue (21, 24, 29). However, in contrast to the data from other countries, the use of OTC medicine during the pandemic was lower than its usage during the pre-pandemic period in Japan (30). Owing to the limited comprehension of the relationship between the use of OTC medicine and digital experience design, it is necessary to continue to study the relationship between digital experience design and Japanese consumer behavior in the OTC medicine purchase process.

To understand Japanese consumers' purchasing behaviors toward OTC medicine and eHealth literacy, digital experience design should be optimized to reduce the potential health risks of inappropriate medication caused by inadequate information and literacy (31–33). Moreover, heath literacy may be a significant variable in determining medical treatment and decision-making (34, 35). eHealth refers to delivering health services and information through the Internet and related technologies (36, 37). eHealth literacy is the ability to utilize digital health information to solve problems regarding health (38); most importantly, it requires the ability to evaluate whether the information on the Internet and other electronic sources is true or false (39). eHealth literacy is essential in digital healthcare (10, 33), and some researchers have found it to be closely related to Japanese consumers' health behavior (40). Meanwhile, for digital experience design, a previous study proposed an Internet-based design to help users search for OTC medicine for self-medication and it yielded a positive result (41). Similarly, during the COVID-19 pandemic, the digital experience design through virtual pharmacist interventions achieved significant outcomes and reduced OTC medicine abuse (42).

Consequently, this study aims to optimize digital experience design for OTC medicine purchases based on comprehending Japanese consumers' current behavior, preferences, eHealth literacy, and their relationships. To this end, the following research questions are formulated:

1. What are consumers' current behavior and preferences while purchasing OTC medicine?
2. What digital experience designs would help consumers to better purchase OTC medicine?
3. How does eHealth literacy relate to the OTC medicine purchasing behavior of consumers?

## 2. Methods

### 2.1. Participants

We conducted an online survey in the Greater Tokyo Area of Japan, which consists of seven prefectures or regions centered in Tokyo Metropolis, on February 2022. This area is one of the leading regions in Japan's economic and technological development; conducting basic research regarding the digital experience design of OTC medicine here is representative. We referred to the age brackets of participants from previous studies and considered potential Internet-based device usage experiences (2, 40, 43). The target respondents were men and women aged 20–49 years. We considered an online survey as appropriate for this study because the respondents can use the Internet successfully. Meanwhile, considering the potential influence of medical knowledge, this recruitment excluded groups of medical-related occupations (3). Freeasy, a leading online survey platform in Japan, recruited participants willing to take the survey from the registered panels, with equal proportions of age brackets and gender, respectively. Participants received points that could be used in place of money at certain stores as compensation for participating in the survey (44, 45). In total, 450 Japanese participants responded to the online survey. Participants were aged from 20 to 29 (*N* = 150), 30 to 39 (*N* = 150), and 40 to 49 (*N* = 150) years old, and 50% (225) were men. The response rate was 100%.

This study employed a screening question and recorded the time it took respondents to complete the survey as evaluation criteria to rule out random and implausibly quick responses. The screening question was, “Which of the following questions is not mentioned in this survey, (i) how to purchase medicines, (ii) methods of collecting medical information, (iii) annual consumption of medicines, and (iv) medication guidance by pharmacists.” Those who chose option (iii) were not excluded. Regarding response time, we calculated that completing the survey would take about 1 min and 40 s without adequately reviewing the contents. Therefore, we used this as another criterion to exclude implausibly fast responses. The data of 288 respondents were finally analyzed.

To detect a correlation coefficient of *r* = 0.21 with 80% power (*α* = 0.05, two-tailed), which is an average effect size in social psychology (46, 47), G*Power suggested we would need 175 participants (47, 48). Meanwhile, 288 valid responses in this study would be sensitive to the effect size *r* = 0.16 with 80% power, the study would be able to reliably detect correlations bigger than *r* = 0.16, which constituted a reasonable sample size (49, 50). In addition, the estimated minimum sample size of other statistical methods did not exceed 175 participants, and the corresponding actual effect size was reported.

### 2.2. Measures

The questionnaire included the following four components: (i) the introduction of the survey; (ii) questions regarding behavior and preferences for OTC medicine purchase; (iii) Japanese version of the eHealth Literacy Scale (J-eHEALS) (51); and (iv) demographic questions, such as age and gender. Research questions were answered through the results of components (ii)–(iv) (see Supplementary Material).

#### 2.2.1. Introduction of the survey

Prior to the survey, we briefed the participants about OTC medicine and informed them about the purpose of this anonymous survey, ethics, institutions, and the researchers involved. The participants were assured that they can withdraw from this survey at any time. Meanwhile, they were asked to follow these guidelines: (i) answer questions alone, without consulting anyone else; (ii) should not eat or drink while answering the questionnaire; (iii) answer the questionnaire in a quiet room without music, TV, etc.

#### 2.2.2. Behavior and preferences regarding OTC medicine purchase

This study attempted to examine the behavior and preferences of respondents in three parts.

The first part focused on the behavior of accessing OTC medicine using the following categorical questions: (i) how do you usually purchase OTC medicines? The answers were “purchase at pharmacies or drug stores,” “purchase on the Internet,” “Other (e.g., convenience stores),” and “I have not purchased OTC medicines”. (ii) Multiple choice questions about the reasons for the specific approach above. (iii) How do you choose OTC medicines? Respondents were asked to rate each given of response from “very often” = 4 to “never” = 0: “choose based on my own experiences,” “choose based on the advice of a pharmacist,” “choose by consulting my family doctor,” “choose after searching for information on the Internet,” “choose based on advice from family and friends”. Cronbach's alpha was 0.74. (iv) In-store, which do you think is a better way to select OTC medicine: directly from the shelves or by searching on the screen of a computer or tablet device?” Respondents were asked to provide details in a text box.

The second part focused on the preferred mode of receiving medical guidance. Respondents were asked to answer, “What is the best way to communicate with a pharmacist to get guidance on medicine: in-person at a pharmacy, online, and either”, and to give a detailed reason in the space provided.

The last part focused on obtaining information on OTC medicine, where respondents were asked the following questions: (i) how often do you get information about OTC medicine from the sources below? Respondents were asked to evaluate TV advertisement, newspaper advertisement, magazine advertisement, Internet advertisement, website of a pharmaceutical manufacturer, Internet search engines (e.g., Yahoo! and Google), side effect database of the Pharmaceuticals and Medical Devices Agency (PDMA), academic societies (e.g., Japan Pharmaceutical Association), private sector (e-pharma), pharmacists, doctors, friends, and family, on a scale of “Very often” = 4 to “Never” = 0. Cronbach's alpha was 0.90. (ii) Have you ever used a smartphone to collect information when purchasing OTC medicine at stores such as local pharmacies and drug stores? The response also ranged “Very often” = 4 to “Never” = 0.

#### 2.2.3. eHealth literacy

eHealth literacy of participants was measured using J-eHEALS (51), which is the Japanese version of the eHealth Literacy Scale (eHEALS) (52) and contained eight questions. Responses were recorded on a 5-point Likert scale from “strongly agree” = 5 to “strongly disagree” = 1. The total score for J-eHEALS ranges from 8 to 40, with a higher score indicating better eHealth literacy. Cronbach's alpha was 0.93 in the present study.

#### 2.2.4. Statistical analyses

Descriptive statistics on the characteristics of participants, including frequency and percentage for categorical variables and mean and standard deviation for continuous variables (mean ± SD), were summarized. Statistical analyses were conducted based on the research questions, with the demographic variables as supplementary analyses.

The outcome for J-eHEALS was calculated as both continuous and categorical variables (53). For J-eHEALS scores, participants were divided into two categories (high or low literacy) relative to the median group value (median 23.00, IQR: 18.00–27.00) based on previous studies (40, 54–56).

The Chi-square, *T*-test, and one-way ANOVA were used to examine the differences among variables. The relationships among eHealth literacy, preferences, and behaviors were analyzed by Pearson correlation tests. IBM SPSS Statistics for MAC, Version 28.0 (Armonk, NY: IBM Corp.) was used for the above statistical analysis, and *p* < 0.05 was considered statistically significant. The effect size for the Chi-square goodness of fit, Chi-square crosstab, *T*-test, and ANOVA was reported as *Cohen's W*, *Cramér's V*, *Cohen's d*, and *η*^2^, respectively (57–59).

In addition, this study used the text-mining approach to comprehend consumer behavior and preferences supplementarily. The text-mining analysis was conducted using KH Coder (60, 61), which enables researchers to keep track of the most frequently used terms, spot word relationships, and organize words in sensible clusters (62, 63). For accurate results, we used KH Coder's programming function to merge close synonyms based on the meaning of original sentences before formally analyzing the text content; for instance, we combined “buy” and “purchase” into a “purchase”, and the “medicine”, “medical product” and “product” were combined into a “medicines” for the analysis.

## 3. Results

The results of research question one were presented in three sections: (i) current behaviors and preferences regarding access to OTC medicine; (ii) preferred mode of receiving medical guidance; (iii) behaviors regarding obtaining OTC medicine information. Research question two was discussed from the results of research question one. The results of research question three were presented as independent variables in the above three sections.

In addition, the characteristics of the participants are shown in Table 1. For eHealth literacy, no significant difference was found between genders, *t*(265.28) = −0.92, *d* = 0.04, *p* = 0.36, and among age brackets, *F*(2, 285) = 2.01, *η*^2^ = 0.01, *p* = 0.14.

**Table 1:** Characteristics of participants.

	Respondents % (*N*)	J-eHEALS (SD)	Mean age (SD)
**Gender**			
Women	52.08% (150)	22.27 (7.30)	34.7 (8.81)
Men	47.92% (138)	22.99 (5.97)	35.78 (8.89)
**Age brackets (years)**			
20–29	32.99% (95)	21.57 (6.40)	24.81 (2.90)
30–39	30.55% (88)	23.44 (6.99)	32.94 (2.94)
40–49	36.46% (105)	22.95 (6.48)	44.86 (2.93)
Total	100.00% (288)	22.65 (6.64)	35.22 (8.85)

### 3.1. Current behavior and preferences regarding access to OTC medicine

Approximately 65.97% (190) of the respondents had the experience of purchasing OTC medicine, 89.47% (170) of whom preferred to buy medicines at local pharmacies or drug stores, 9.47% (18) preferred to purchase online, and 1.05% (2) chose the option “others,” *χ*^2^(3) = 251.33, *W* = 0.93, *p* < 0.001. There was no significant difference in the choice of offline or online purchasing between participants with high and low eHealth literacy, *χ*^2^(1) = 0.44, *V* = 0.04, *p* = 0.51, men and women, *χ*^2^(1) = 1.05, *V* = 0.06, *p* = 0.31, and the different age brackets, *χ*^2^(2) = 2.12, *V* = 0.06, *p* = 0.35. For respondents' specific reasons for purchasing OTC medicine, the results are shown in Table 2.

**Table 2:** Specific reasons for purchasing OTC medicine.

Respondents % (*N*)	Purchase OTC medicines at local pharmacies or stores	Purchase OTC medicines online	Others
Safety	41.18% (70)	22.22% (4)	–
Privacy	5.88% (10)	27.78% (5)	–
Reliability	24.12% (41)	5.56% (1)	–
Anonymity	7.65% (13)	5.56% (1)	–
Communication with pharmacist	25.29% (43)	16.67% (3)	–
Guidance for intake	9.41% (16)	11.11% (2)	–
24-hour availability	14.12% (24)	55.56% (10)	–
Wide-spread availability	46.47% (79)	50.00% (9)	–
Convenience	40.59% (69)	44.44% (8)	–
Others	1.18% (2)	11.11% (2)	–
Total	89.47% (170)	9.47% (18)	1.05% (2)

This survey question was linked to the previous question, “How do you usually purchase OTC medicines?” The sample size for this table is 190 participants with the OTC medicine purchasing experience.

The results regarding opinions on the preferred approach to choosing OTC medicine at local pharmacies or drug stores, as seen in Table 3, implied that most of respondents accepted to choose medicines on the shelf and digital screen, *χ*^2^(3) = 102.36, *W* = 0.60, *p* < 0.001. Moreover, “searching on-screen” showed a slightly better acceptance in consumers with high eHealth literacy, *χ*^2^(3) = 10.26, *V* = 0.19, *p* = 0.02. There were no significant differences between genders, *χ*^2^(3) = 2.75, *V* = 0.10, *p* = 0.44, and among different age brackets, *χ*^2^(6) = 5.05, *V* = 0.09, *p* = 0.55.

**Table 3:** Preferred approaches to choosing OTC medicine.

Respondents % (*N*)	Selecting on the shelf	Searching on-screen	Either	Neither
**eHealth literacy** *				
High J-eHEALS	29.50% (41)	27.34% (38)	41.72% (58)	1.44% (2)
Low J-eHEALS	34.90% (52)	14.09% (21)	45.64% (68)	5.37% (8)
**Gender**				
Women	28.00% (42)	22.00% (33)	46.00% (69)	4.00% (6)
Men	36.96% (51)	18.84% (26)	41.30% (57)	2.90% (4)
**Age brackets (years)**				
20–29	27.37% (26)	23.16% (22)	45.26% (43)	4.21% (4)
30–39	40.90% (36)	15.90% (14)	39.77% (35)	3.41% (3)
40–49	29.52% (31)	21.90% (23)	45.71% (48)	2.86% (3)
Total**	32.29% (93)	20.49% (59)	43.75% (126)	3.74% (10)

* Significant difference at the 0.05 level (2-tailed).

** Significant difference at the 0.001 level (2-tailed).

**Figure 1:**
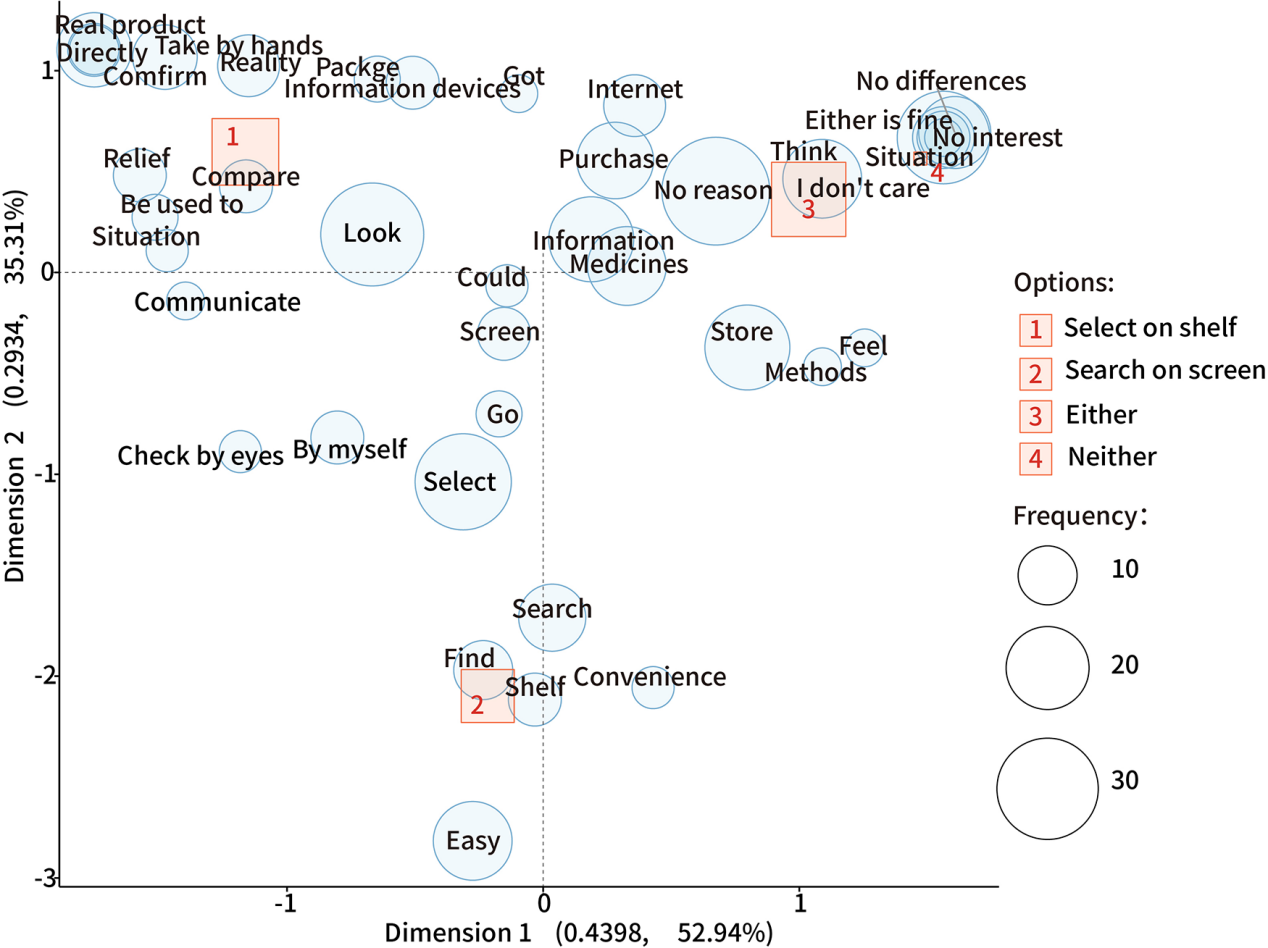
The correspondence analysis for the specific approach to choosing OTC medicine. In this plot, uncharacteristic words uniformly found in all options are plotted near the origin (0,0); a closer distance between a word and an option indicates a more specific association; the further away from the origin, the more characteristic the word is and is distinguished from the other options.

Regarding why respondents chose a specific approach to select OTC medicine, the text-mining analysis shown in Figure 1, suggested that the reasons to select from the shelves were to be able to directly pick up the medicines by hand, ease in comparing and confirming, and relief of communicating (with the pharmacist) and checking the actual product. For those who preferred to search for medicines on-screen, they found it easy and convenient to find the medicines and medical information. Meanwhile, those who chose either approach preferred to choose a specific approach based on their circumstances but were fine with either. In addition, a few respondents said, “searching for information using a screen was a good experience but getting medicines was not,” “I need help from the pharmacists at the stores,” “I cannot find and read the medical information on a screen,” and “I search and check the medical information and feedback online before going to a pharmacy.”

**Figure 2:**
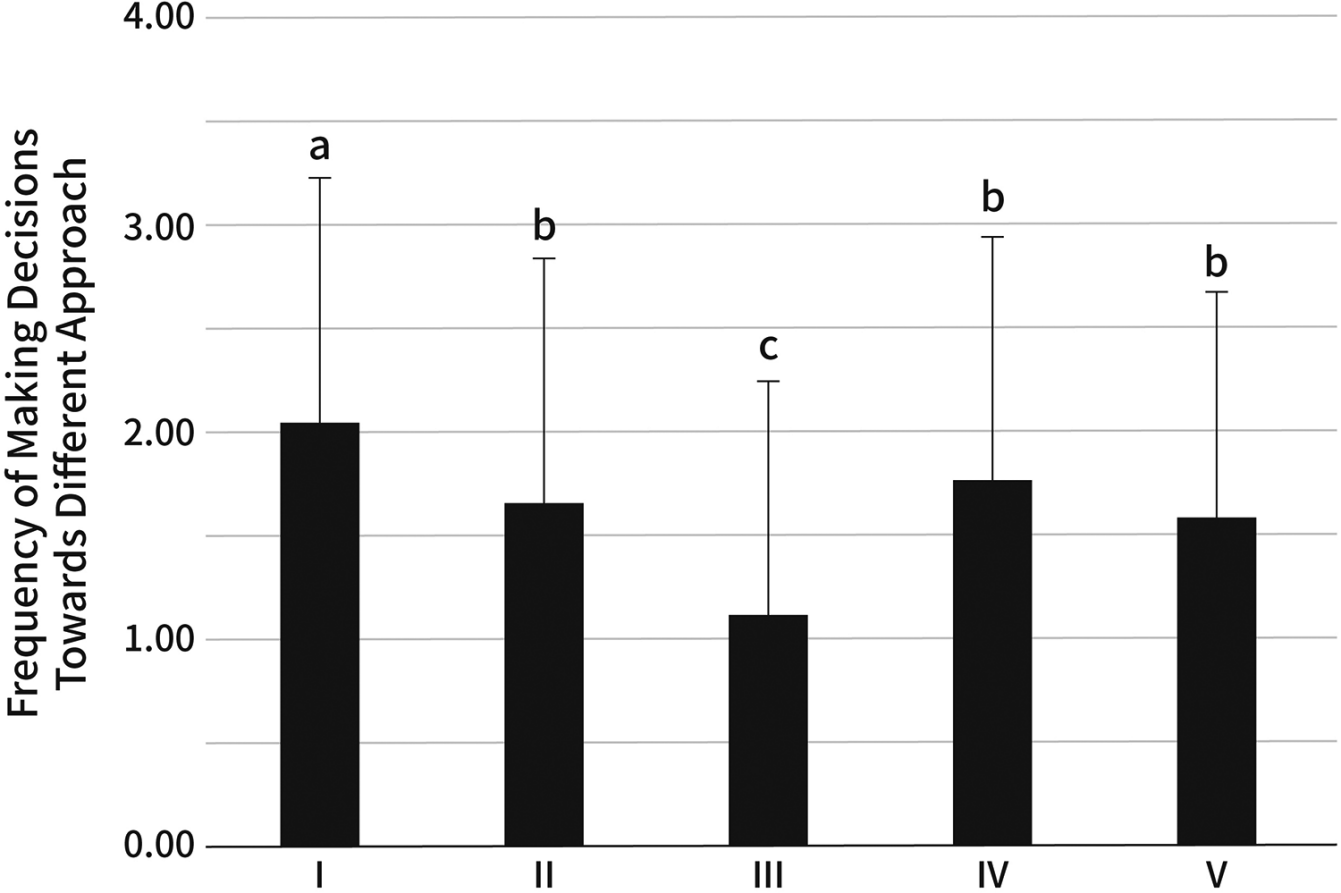
Frequency of approaches in making the decision to choose OTC medicines. The approaches are: (I) own experiences (2.02 ± 1.20), (II) advice from a pharmacist (1.65 ± 1.19), (III) consulting a family doctor (1.10 ± 1.14), (IV) searching for information on the Internet (1.75 ± 1.18), (V) advice from family and friends (1.57 ± 1.11). The same letter indicates that the difference is not significant (*p* > 0.05), and different letters indicate a significant difference (*p* < 0.05).

With regard to how a decision to choose OTC medicine is made, the one-way ANOVA showed significant differences among the approaches, *F*(4, 1,435) = 23.77, *η*^2^*^ ^*= 0.06, *p* < 0.001. The Tukey honestly significant difference (HSD) results for multiple comparisons are shown in Figure 2. Purchasing OTC medicines based on consumers' own experiences was significantly higher than choosing based on advice from a pharmacist, family doctor, the Internet, and family and friends.

Meanwhile, the Pearson correlation test showed significant positive correlations between the approaches to making a decision and J-eHEALS scores (Table 4). More specifically, searching for information on the Internet was relatively strongly correlated with choosing OTC medicine on one's own experiences and getting advice from family and friends, but weakly correlated with getting advice from a pharmacist and consulting a family doctor. Moreover, a relatively strong correlation was found between getting advice from a pharmacist and a family doctor.

**Table 4:** Correlation between the approaches to making the decision to purchase OTC medicine and eHealth literacy scores.

	1. Own experiences	2. Advice from pharmacist	3. Consulting family Doctor	4. Searching for information on the internet	5. Advice from family and friends
J-eHEALS	.21***	.22***	.17**	.27***	.13*
1		.29***	.06	.55***	.45***
2			.63***	.33***	.38***
3				.25***	.25***
4					.51***

* Correlation is significant at the 0.05 level (2-tailed).

** Correlation is significant at the 0.01 level (2-tailed).

*** Correlation is significant at the 0.001 level (2-tailed).

### 3.2. Preferred mode of receiving medical guidance

The results regarding how medicine guidance was received, as shown in Table 5, *χ*^2^(2) = 47.31, *W* = 0.41, *p* < 0.001. There was no significant difference among the levels of eHealth literacy, *χ*^2^(2) = 2.33, *V* = 0.09, *p* = 0.31, genders, *χ*^2^(2) = 1.34, *V* = 0.07, *p* = 0.51, and different age brackets, *χ*^2^(4) = 2.08, *V* = 0.06, *p* = 0.72, respectively.

**Table 5:** Results regarding how to receive medicine guidance.

Respondents % (*N*)	Face to face at the local pharmacy	Online	Either
**eHealth literacy**			
High J-eHEALS	40.29% (56)	17.27% (24)	42.45% (59)
Low J-eHEALS	46.31% (69)	11.41% (17)	42.28% (63)
**Gender**			
Women	42.00% (63)	12.67% (19)	45.33% (68)
Men	44.93% (62)	15.94% (22)	39.13% (54)
**Age brackets (years)**			
20–29	38.95% (37)	17.89% (17)	45.26% (41)
30–39	46.59% (41)	23.50% (11)	40.91% (36)
40–49	44.76% (47)	12.38% (13)	42.86% (45)
Total*	43.40% (125)	14.24% (41)	42.36% (122)

* Significant difference at the 0.001 level (2-tailed).

**Figure 3:**
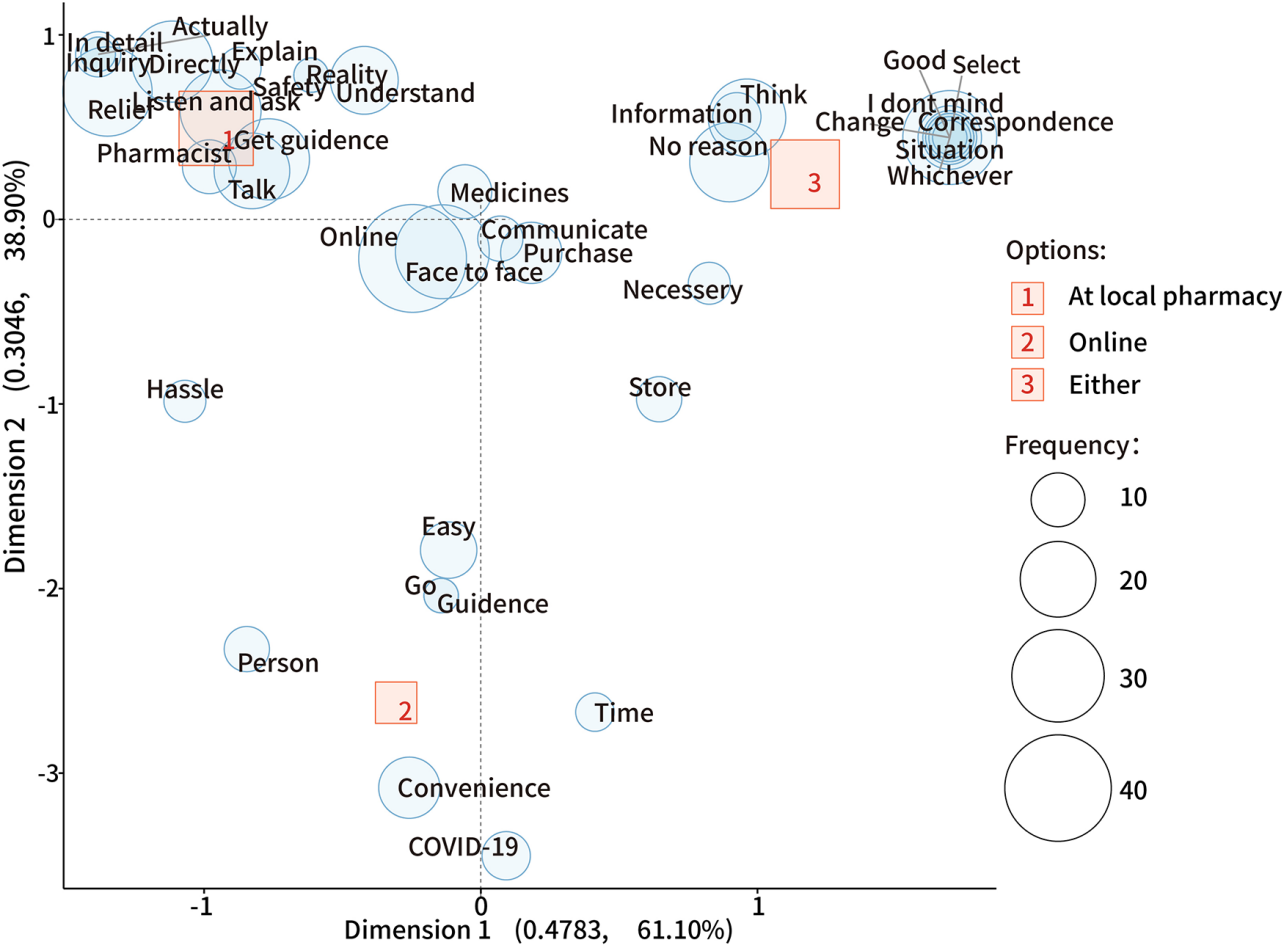
The correspondence analysis for the different choices to receive OTC medication guidance. In this plot, uncharacteristic words uniformly found in all options are plotted near the origin (0,0); a closer distance between a word and an option indicates a more specific association; the further away from the origin, the more characteristic the word is and is distinguished from the other options.

Furthermore, through the text-mining analysis (Figure 3), the main reason for seeking guidance at a local pharmacy was receiving and inquiring about medical information directly, especially detailed explanations, and feeling relieved and safe. Respondents chose online guidance for convenience, effortlessness, ease of finding time to communicate online, and to avoid COVID-19 infection. Respondents accepted both approaches because they liked to have options depending on different situations. Moreover, a few respondents said that, “I cannot understand the medication information without communicating with the pharmacist face-to-face” and “the pharmacist is the same person online and offline.”

### 3.3. Behaviors regarding obtaining OTC medicine information

**Figure 4:**
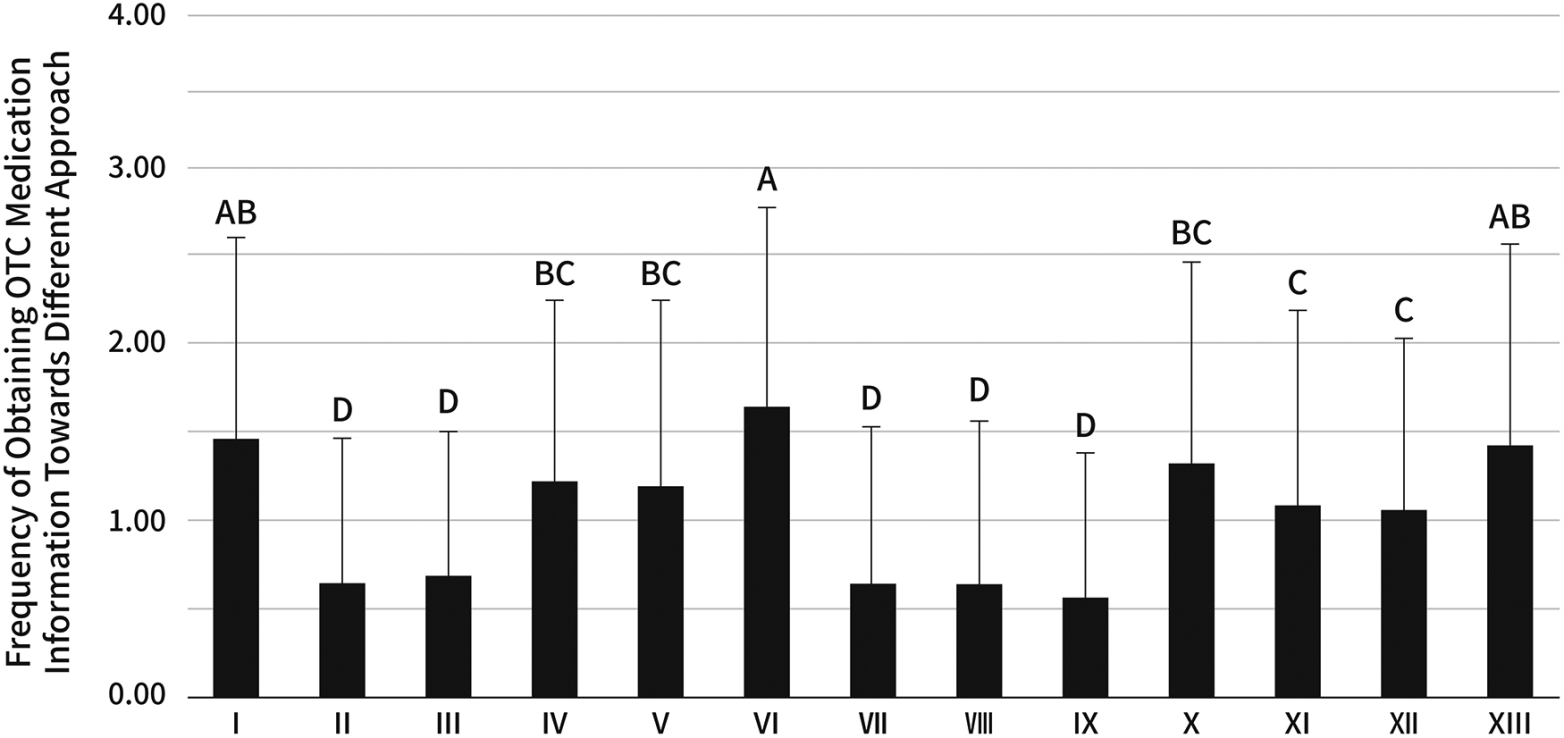
Frequency of approaches preferred in collecting information on OTC medicine. The approaches are: (I) TV advertisement (1.47 ± 1.14), (II) Newspaper advertisement (0.64 ± 0.83), (III) magazine advertisement (0.68 ± 0.81), (IV) Internet advertisement (1.22 ± 1.03), (V) Website of a pharmaceutical manufacturer (1.19 ± 1.05), (VI) Internet search engines (e.g., Yahoo! and Google) (1.65 ± 1.14), (VII) Side effect databases of the PDMA (0.64 ± 0.89), (VIII) Academic societies (e.g., Japan Pharmaceutical Association) (0.65 ± 0.91), (IX) private sector (e-pharma) (0.57 ± 0.81), (X) pharmacists (1.33 ± 1.14), (XI) Doctors (1.10 ± 1.10), (XII) Friends (1.06 ± 0.97), (XIII) Family (1.42 ± 1.15). The same letter indicates that the difference is not significant (*p* > 0.05), and different letters indicate a significant difference (*p* < 0.01).

Regarding gathering information on OTC medicine, one-way ANOVA showed significant differences in mean exam scores between at least of the approaches to collecting information, *F*(12, 3,731) = 39.84, *η*^2^*^ ^*= 0.11, *p* < 0.001. The results of post-hoc Tukey HSD for multiple comparisons are shown in Figure 4. The main approaches for respondents to obtain medical information were Internet search engines (e.g., Yahoo, Google), TV advertisement, and their family members.

Furthermore, the relationships between the eHealth literacy and approaches preferred to collect information were conducted as shown in Table 6. The Pearson correlation test indicated significant positive correlations between J-eHEALS and the approach to obtaining OTC medication information, especially through Internet search engines. Meanwhile, there were very strong correlations among similar behavior in obtaining medical information. For obtaining information from advertisements, relatively strong correlations were found among obtaining information through TV, newspaper, magazine, and the Internet. Moreover, for collecting information from agencies, there were very strong correlations between PDMA and academic societies; and between PDMA and private sectors. For receiving information from people, a very strong correlation was found between pharmacists and family doctors, and a relatively strong correlation was found between friends and family.

**Table 6:** Correlations between behavior in obtaining OTC medicine information and eHealth literacy scores of consumers.

	1. TV advertisement	2. Newspaper advertisement	3. Magazine advertisement	4. Internet advertisement	5. Website of a Pharmaceutical manufacturer	6. Internet search engines	7. PDMA	8. Academic societies	9. Private sector	10. Pharmacists	11. Doctors	12. Friends	13. Family
J-eHEALS	.28***	.25***	.243***	.23***	.29***	.37***	.24***	.26***	.32***	.19**	.17**	.25**	.14*
1		.47***	.45***	.58***	.40***	.40***	.19**	.19**	.21***	.33***	.26***	.52***	.49***
2			.76**	.45**	.46**	.25**	.55**	.54**	.56**	.27**	.38**	.38**	.23**
3				.61***	.52***	.32***	.59***	.54***	.55***	.40***	.42***	.46***	.25***
4					.56***	.42***	.30***	.29***	.33***	.40***	.38***	.54***	.38***
5						.55***	.55***	.54***	.50***	.45***	.41***	.39***	.24***
6							.31***	.25***	.33***	.33***	.27***	.39***	.37***
7								.82***	.77***	.36***	.41***	.29***	.20**
8									.69***	.37***	.40***	.31***	.17**
9										.37***	.40***	.31***	.16**
10											.70***	.45***	.39***
11												.43***	.37***
12													.67***

* Correlation is significant at the 0.05 level (2-tailed).

** Correlation is significant at the 0.01 level (2-tailed).

*** Correlation is significant at the 0.001 level (2-tailed).

Regarding collecting medical information at local pharmacies and stores, as shown in Table 7, 85.26% (162) of the participants who had the experience of purchasing OTC medicine reported using a smartphone to collect information about OTC medicine at pharmacies or drug stores, *χ*^2^(4) = 53.63, *W* = 0.53, *p* < 0.001. One-sample *T*-test (compared to “occasionally” values) showed a relatively high frequency of smartphone use at local pharmacies or drug stores (1.81 ± 1.14), *t*(189) = 9.73, *d* = 0.71, *p* < 0.001. The results implied that participants with high literacy have a higher proportion of smartphone use at pharmacies or stores, *χ*^2^(4) = 17.65, *V* = 0.31, *p* = 0.001. Meanwhile, participants aged from 20 to 29 had a slightly higher proportion of smartphone use as well, *χ*^2^(8) = 19.49, *V* = 0.23, *p* = 0.01. No significant difference was found between genders, *χ*^2^(4) = 5.83, *V *= 0.18, *p* = 0.21.

**Table 7:** Results of using smartphones to collect information about OTC medicine at pharmacies or drug stores.

Respondents % (*N*)	Very often	Often	Sometimes	Occasionally	Never
**eHealth literacy** **					
High J-eHEALS	8.74% (9)	19.24% (20)	46.60% (48)	16.50% (17)	8.74% (9)
Low J-eHEALS	10.34% (9)	6.90% (6)	31.03% (27)	29.89% (26)	21.84% (19)
**Gender**					
Women	8.57% (9)	19.05% (20)	37.14% (39)	21.90% (23)	13.13% (14)
Men	10.59% (9)	7.06% (6)	42.35% (36)	23.53% (20)	16.47% (14)
**Age brackets (years)** *					
20–29	16.67% (11)	15.15% (10)	50.00% (33)	12.12% (8)	6.06% (4)
30–39	7.41% (4)	11.11% (6)	35.19% (19)	25.93% (14)	20.37% (11)
40–49	4.29% (3)	14.29% (10)	32.86% (23)	30.00% (21)	18.57% (13)
Total***	9.47% (18)	13.68% (26)	39.47% (75)	22.63% (43)	14.74% (28)

* Significant difference at the 0.05 level (2-tailed).

** Significant difference at the 0.01 level (2-tailed).

*** Significant difference at the 0.001 level (2-tailed).

## 4. Discussion

To answer the research questions, this study used quantitative and text-mining research methods to try to optimize digital experience design regarding OTC medicine purchases based on comprehending Japanese consumers' current behavior, preferences, eHealth literacy, and their relationships. The research questions were answered in three sections: (i) behavior and preferences regarding OTC medicine purchase; (ii) association between eHealth literacy and OTC purchasing behaviors; (iii) opportunities and risks for hybrid digital experience design.

### 4.1. Behavior and preferences regarding OTC medicine purchase

Purchasing OTC medicine in-store is the primary approach for Japanese consumers. This behavior is consistent with previous research findings (18, 19). Moreover, our results suggest that even during the pandemic, most Japanese consumers still prefer to buy medicine at local pharmacies or stores due to safety, reliability, convenience, and communication with pharmacists. Furthermore, choosing medicines from the shelf is the first choice for Japanese consumers; however, selecting medicines through a digital screen did not indicate a rejection.

Consumers gather medical information from different sources to make decisions. Our results indicate that making decisions based on self-experience has a relatively strong correlation with collecting information on the Internet, which could be one of the primary sources for solitary decision-making. This phenomenon may be related to their ability to search for information on the Internet and their Internet dependence (64). It is worth noting that consumers accustomed to relying on professional advice may not trust the information online. We found a relatively strong correlation between depending on the advice of professionals, such as in making purchasing decisions and obtaining medical information from pharmacists and doctors; in contrast, a weaker correlation was found between relying on professional guidance and obtaining information via the Internet for decision making.

Regarding medicine guidance, this study revealed that most consumers prefer receiving guidance face-to-face from a pharmacist in-store due to the convenience and comfort of direct communication. On the other hand, accepting guidance online also has advantages, such as ease, freedom to ask, and reduced exposure to COVID-19 during the pandemic.

Japanese consumers show a slightly high frequency of willingness to obtain medical information through medical personnel. However, our results indicate that most of them may not value information on OTC medicine received through official informatic channels. Internet search engines, TV commercials, and family recommendations were the primary sources for consumers to obtain the information. Previous studies have shown that only 39% of websites provide accurate health information, and these are primarily government-created (65, 66). It is essential to be wary of health misinformation on the Internet (67, 68). Moreover, it is also necessary to be vigilant about consumers who may not be able to judge the information accurately from TV advertisements (2).

Moreover, Japanese consumers frequently use smartphones to find OTC medicine information in local pharmacies and drug stores, which indicates that they require additional medical information aside from the information on packages. Younger consumers have a slightly higher frequency of searching for medical information on smartphones than consumers in other age brackets. This may be derived from the behavior of young consumers who are more prone to use the Internet to find health information (33, 69).

### 4.2. Correlation between OTC purchasing behaviors and eHealth literacy

The average score for eHealth literacy of Japanese consumers in the present study is lower than that in the previous studies (40, 51, 70).

eHealth literacy is more associated with consumers' digital behaviors toward OTC medicine information acquisition but less associated with medicine purchases and selections. Our results suggest that the associations between behaviors are higher compared with eHealth literacy and behaviors regarding OTC medicine. Consumers with high eHealth literacy indicate a slightly higher preference for choosing medicine through digital devices, and they are more inclined to obtain medical information through the Internet, particularly the smartphone.

### 4.3. Opportunities and risks for hybrid digital experience design

The digital experiences design for OTC medicine purchases could not be purely in-store or online; it needs to be based on consumer behavior. Combining the text-mining analysis suggests that an Internet-based design could provide more medical information to help in decision-making even though the design does not offer a sense of realism, comfort, and safety. This phenomenon could be explained by Internet-based experience design not providing the same sense of immediacy, security, and reliability as offline pharmacies. However, it can provide more prosperous and timely medical information to help self-health management (71).

The present study proposes a hybrid design concept based on consumer behavior while corroborating the conceptual assumptions of the previous research (17). Delivering a hybrid digital experience may be a logical and appropriate approach to accessing reliable medical information and making appropriate decisions. Meanwhile, the mobile digital experience should be considered; our results show a migration to smartphone-linked health behavior at pharmacies or stores. While utilizing the benefits of digitization, it is crucial to alleviate the anxiety and skepticism brought on by digital design from the viewpoint of consumers and offer them an experience that is comforting, safe, reliable, understandable, and effective.

Hence, the digital experience design in-store vs. online could play different functional roles. The digital experience in-store could focus on meeting the needs of various consumers, especially through an expert's direction on how to personalize and efficiently choose medicine and get guidance considering digital advantages. Whereas, the online digital experience design, not only for traditional Internet services but also for mobile Internet, could focus on consumers accessing medicine, especially the necessary information, efficiently: including security, legibility, and reliability. Additionally, ensuring consistency in information design and interaction logic between in-store and online may be a critical factor in bridging the gap between the in-store and the online experience. Taking into account the potential risks of the behavior of consumers and the effects of poor eHealth literacy, simply digitally transforming pharmacies in Japan would not be feasible.

## 5. Limitations

This online survey has several limitations. First, the demographics of the participants recruited for this study were not sufficiently comprehensive. This study did not consider factors such as income and education. Moreover, the elderly group was not taken into consideration, as Japan's aging problem may affect the retail industry (72). Therefore, considering the potential difference in behavior (73), there is a need for a separate in-depth study on digital experience design for the elderly. Second, there is a sample size bias. This survey was only conducted in the Great Tokyo of Japan. The impact of the knowledge of healthcare professionals was also excluded; therefore, it is still necessary to appropriately expand the research scope in future studies. Third, some behaviors and preferences were not adequately explained, as the study only focused on consumer behavior and preferences from the perspective of digital experience design. It is necessary to combine the interactive prototype design to deeply understand the potential impact of digital design on consumer behavior. Finally, as an online study, this study only puts forward a preliminary conception of hybrid digital experience design regarding OTC medicine purchases. The results of this study could not support the proposal of specific design models for the time being, which necessitates further research.

## 6. Conclusions

Japanese consumers are trying to find a combination of conventional and digital behaviors for purchasing OTC medicine rather than opting for a particular method. Most consumers prefer purchasing and receiving instructions in-store while searching for additional decision-making information online. eHealth literacy is positively associated with digital behaviors of OTC medicine information acquisition but less associated with medicine purchases and selections. The hybrid digital experience design may enhance the experience regarding OTC medicine purchases and reduce potential risks by providing information appropriately.

## Data Availability

The raw data supporting the conclusions of this article will be made available by the authors, without undue reservation.
