# Supplementary material for: Analysis of Japanese consumers' attitudes toward the digital transformation of OTC medicine purchase behavior and eHealth literacy: an online survey for digital experience design
Source: Front Digit Health. 2023 May 24;5:1173229. doi: 10.3389/fdgth.2023.1173229 (PMC10244771; doi:10.3389/fdgth.2023.1173229)
Supplement: Supplementary file 1 [file Datasheet1.pdf]

Supplementary Material

1 List of survey questions

Behaviors and preferences regarding OTC medicine purchase	
Part 1: Behavior of accessing OTC medicine	(i) How do you usually purchase OTC medicines?<br>- Purchase at pharmacies or drug stores<br>- Purchase on the Internet<br>- Other (e.g., convenience stores)<br>- I have not purchased OTC medicines<br><br>(ii) Multiple choice questions about the reasons for the specific approach above (linked to question (i)).<br>- Safety<br>- Privacy<br>- Reliability<br>- Anonymity<br>- Communication with pharmacist<br>- Guidance for intake<br>- 24-hour availability<br>- Wide-spread availability<br>- Convenience<br>- Others<br><br>(iii) How do you choose OTC medicines? Respondents were asked to rate each given of response from "very often" = 4 to "never" = 0<br>- Choose based on my own experiences<br>- Choose based on the advice of a pharmacist<br>- Choose by consulting my family doctor<br>- Choose after searching for information on the Internet<br>- Choose based on advice from family and friends<br><br>(iv) In-store, which do you think is a better way to select OTC medicine: directly from the shelves or by searching on the screen of a computer or tablet device?" Respondents were asked to provide details in a text box.
Part 2: Preferred mode of receiving medical guidance	(i) What is the best way to communicate with a pharmacist to get guidance on medicine?<br>- In-person at a pharmacy<br>- Online<br>- Either

	(ii)	What is your reason? Respondents were asked to provide details in a text box.
Part 3: Obtaining information on OTC medicine	(i)	How often do you get information about OTC medicine from the sources below? Respondents were asked to rate each given of response from “very often” = 4 to “never” = 0.<br>- TV advertisement<br>- Newspaper advertisement<br>- Magazine advertisement<br>- Internet advertisement<br>- Website of a pharmaceutical manufacturer<br>- Internet search engines (e.g., Yahoo! and Google)<br>- Side effect database of the Pharmaceuticals and Medical Devices Agency (PDMA)<br>- Academic societies (e.g., Japan Pharmaceutical Association)<br>- Private sector (e-pharma)<br>- Pharmacists<br>- Doctors<br>- Friends<br>- Family
	(ii)	Have you ever used a smartphone to collect information when purchasing OTC medicine at stores such as local pharmacies and drug stores?<br>Respondents were asked to rate each given of response from “very often” = 4 to “never” = 0.
**eHealth literacy**		
Japanese version of the eHealth Literacy Scale (J-eHEALS)		
**Demographic questions**		
Age and Gender		
